# The Hydriatic Treatment of Tuberculosis

**Published:** 1900-12-15

**Authors:** 


### THE HYDRIATIC TREATMENT OF TUBERCULOSIS.

DR. J. H. KELLOGG,[1] of Battle Creek, writes in high praise of what he calls the hydriatic treatment of tuberculosis. Basing his proceedings on the fact that the power of healing all disease lies in the body itself, he endeavours to bring into play all the resources of hydrotherapy so as to "build up the person's vital resistance, to improve the quality of his tissues, to raise his whole vital status, and to recover as far as possible his original ability to resist the invasion of parasitic organisms and to destroy them when they have gained entrance to the body." A worthy ambition. Dr. Kellogg fully accepts modern views about the advantage of open air, pure air, mountain air, &c., but he finds that in addition to all this much can be done by hydriatic treatment, especially in the hot months of the summer season, which, as he truly says, are often extremely trying to the tuberculous patient. The method consists of a somewhat elaborate use of the "rubs" and "tubs" and "packs" and "sheets" in common use at many "hydros," and of various special applications chiefly in the form of "packs" applied locally. Dr. Kellogg gives some very good cases, and we may say at once that there is much reason for believing that there is a considerable field for hydro-therapy both in the general and in the symptomatic treatment of consumption, although it must be remembered that the "water cure" in such cases is rather a double-edged weapon, and one which requires constant professional supervision. Many of the measures recommended might be found difficult to carry out efficiently except in an institution provided with trained attendants, but the employment of the chest-pack as a means of relieving cough, of which Dr. Kellogg speaks in the highest praise, may be found of very general utility. He says: "Patients who have been almost entirely deprived of rest by a troublesome night cough are often at once so relieved that abundance of refreshing sleep is obtained by the simple application of the chest-pack at night. In cases in which the circulation is feeble the wet towel should be covered with oiled muslin, mackintosh, or gutta-percha tissue, in addition to the flannel wrappings." Dr. Kellogg by no means limits the utility of hydriatic treatment to early cases, holding that in most unpromising cases with large cavities a marvellous degree of improvement is often secured; but it must be borne in mind that he considers residence in a dry, elevated region a condition essential for the best results in the majority of cases.

[1] New York Med. News, Nov. 10, 17, and 24.

